# Spatial clustering of drug-resistant tuberculosis in Hlabisa subdistrict, KwaZulu-Natal, 2011–2015

**DOI:** 10.5588/ijtld.17.0457

**Published:** 2018-03

**Authors:** C. M. Smith, R. Lessells, A. D. Grant, K. Herbst, F. Tanser

**Affiliations:** * Centre for Public Health Data, Institute of Health Informatics, University College London, London; † Department of Clinical Research, London School of Hygiene & Tropical Medicine, London, UK; ‡ Africa Health Research Institute, School of Nursing and Public Health, University of KwaZulu-Natal, Somkhele; § School of Public Health, University of the Witwatersrand, Johannesburg; ¶ School of Nursing and Public Health, University of KwaZulu-Natal, Durban; # Centre for the AIDS Programme of Research in South Africa, University of KwaZulu-Natal, Congella, South Africa

**Keywords:** geographic information systems, disease clustering, HIV infection

## Abstract

**SETTING:**

Incidence rates of tuberculosis (TB) in South Africa are among the highest in the world, and drug resistance is a major concern. Understanding geographic variations in disease may guide targeted interventions.

**OBJECTIVE:**

To characterise the spatial distribution of drug-resistant TB (DR-TB) in a rural area of KwaZulu-Natal, South Africa, and to test for clustering.

**DESIGN:**

This was a cross-sectional analysis of DR-TB patients managed at a rural district hospital from 2011 to 2015. We mapped all patients in hospital data to local areas, and then linked to a population-based demographic surveillance system to map the patients to individual homesteads. We used kernel density estimation to visualise the distribution of disease and tested for clustering using spatial scan statistics.

**RESULTS:**

There were 489 patients with DR-TB in the subdistrict; 111 lived in the smaller demographic surveillance area. Spatial clustering analysis identified a high-risk cluster (relative risk of DR-TB inside vs. outside cluster 3.0, *P* <0.001) in the south-east, a region characterised by high population density and a high prevalence of human immunodeficiency virus infection.

**CONCLUSION:**

We have demonstrated evidence of a geographic high-risk cluster of DR-TB. This suggests that targeting interventions to spatial areas of highest risk, where transmission may be ongoing, could be effective.

## Abstract (FR)

**CADRE:**

Les taux d’incidence de la tuberculose (TB) en Afrique du Sud sont parmi les plus élevés dans le monde et la pharmacorésistance est une préoccupation majeure. Comprendre les variations géographiques de la maladie pourrait guider des interventions ciblées.

**OBJECTIF:**

Nous avons voulu caractériser la distribution spatiale de la TB pharmacorésistante (TB-DR) dans une zone rurale du KwaZulu-Natal, Afrique du Sud, et tester les regroupements.

**SCHÉMA:**

Ceci a été une analyse transversale de patients TB-DR pris en charge dans un hôpital de district rural de 2011 à 2015. Nous avons réalisé une cartographie de tous les patients à partir des données de l’hôpital vers les zones locales, et nous les avons liées à un système de surveillance démographique en population afin de situer les patients dans leurs fermes respectives. Nous avons utilisé l’estimation à noyau de la densité pour visualiser la distribution de la maladie et testé les regroupements à l’aide de statistiques d’exploration spatiale.

**RÉSULTATS:**

Il y a eu 489 patients atteints de TB-DR dans le sous district ; 111 vivaient dans la zone de surveillance démographique plus petite. L’analyse des regroupements spatiaux a identifié un groupe à haut risque (risque relatif de TB-DR dans le groupe par rapportà l’extérieur : 3,0 ; *P* <0,001) dans le sud-est, une région caractérisée par une grande densité de population et une prévalence élevée du virus de l’immunodéficience humaine.

**CONCLUSION:**

Nous avons apporté la preuve d’un regroupement géographique à risque élevé de TB-DR. Ceci suggère qu’il serait efficace de cibler les interventions sur des zones spatiales du risque le plus élevé, où la transmission peut être continue.

## Abstract (SP)

**MARCO DE REFERENCIA:**

Sudáfrica presenta una de las tasas de incidencia de tuberculosis (TB) más altas del mundo y la farmacorresistencia plantea allí un problema mayor. La comprensión de las variaciones geográficas en la enfermedad puede orientar la ejecución de intervenciones dirigidas.

**OBJETIVO:**

Caracterizar la distribución espacial de los casos de TB farmacorresistente (TB-DR) en una zona rural de KwaZulu-Natal, en Sudáfrica, y analizar la presencia de conglomerados.

**MÉTODO:**

Fue este un análisis transversal de los pacientes con diagnóstico de TB-DR e atendidos en un hospital distrital rural del 2011 al 2015. Todos los pacientes incluidos en los registros hospitalarios se cartografiaron en las zonas locales y se vincularon con un sistema de vigilancia demográfica poblacional, con el fin de localizar la vivienda de cada paciente. Mediante la estimación de las densidades focales (Kernel) se visualizó la distribución de la enfermedad y se analizó la presencia de conglomerados con estadísticas de barrido espacial.

**RESULTADOS:**

Se registraron 489 pacientes con TB-DR en el subdistrito, de los cuales 111 vivían en la zona restringida de vigilancia demográfica. El análisis espacial de conglomerados puso en evidencia un conglomerado de alto riesgo en la región suroriental (riesgo relativo de TB-DR dentro del conglomerado, con respecto a fuera del conglomerado: 3,0; *P* <0,001), una región que se caracteriza por una alta densidad de población y alta prevalencia de infección por el virus de la inmunodeficiencia humana.

**CONCLUSIÓN:**

El presente estudio reveló un conglomerado geográfico con algo riesgo de presencia de TB-DR. Esta situación indica que pueden ser eficaces las intervenciones dirigidas a zonas espaciales de alto riesgo, donde tal vez existe una transmisión activa.

INCIDENCE RATES OF TUBERCULOSIS (TB) in South Africa are among the highest in the world.^1^ In 2015, there were an estimated 454 000 new diagnoses, at a rate of 834 per 100 000 population, making it the leading natural cause of death in the country.^1^,^2^ TB rates are particularly high in the province of KwaZulu-Natal, largely driven by the high prevalence of human immunodeficiency virus (HIV) infection and complicated by anti-tuberculosis drug resistance.^3^,^4^

Understanding the spatial distribution of disease is important for effective control. Spatial analyses can be used to identify the worst affected areas, generate hypotheses about transmission, and guide interventions.^5^ Tests of spatial clustering can be used to identify groups of patients that occur closer together in space than would be expected by chance. These analyses have been used to identify areas of likely TB transmission.^6^–^14^ Visualisation of spatial data on maps also provides a powerful means of communicating information about the disease to policy makers and the public.

The Africa Health Research Institute (AHRI) in the Hlabisa subdistrict of KwaZulu-Natal, South Africa, maintains a large health and demographic surveillance system. This includes individual residential locations mapped to an accuracy of <2 m, and routine linkage to public sector records.^15^,^16^

The aim of the present study was to characterise the spatial distribution of drug-resistant TB (DR-TB) in the subdistrict, test for spatial clustering and discuss implications for prevention and care.

## STUDY POPULATION AND METHODS

### Study area

**Figure 1.**
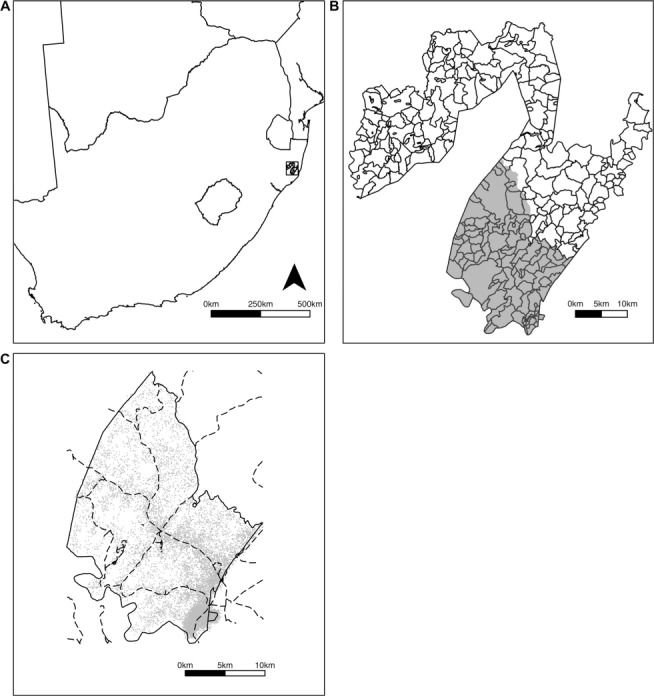
Study site. A) Location of Hlabisa subdistrict in South Africa; B) Hlabisa subdistrict, showing local areas and Africa Health Research Institute demographic surveillance area (shaded); C) Africa Health Research Institute demographic surveillance area, with roads and approximate locations of homesteads (incorporating intentional random error).

The Hlabisa health subdistrict is an area of approximately 1450 km^2^ with 247 350 residents in uMkhanyakude District, northern KwaZulu-Natal (Figure 1A). It is characterised by a high prevalence of HIV infection, with high rates of associated TB (577 recorded TB cases per 100 000 population in uMkhanyakude in 2015; 64.3% HIV-positive).^3^,^17^ The AHRI demographic surveillance area is located within the Hlabisa subdistrict (Figure 1B). This is a region of 435 km^2^, with approximately 11 000 homesteads and 60 000 residents, where the AHRI has been conducting population-based demographic surveillance since 2000.^15^

### Data sources and identification of drug-resistant tuberculosis patients

This was a cross-sectional analysis of patients diagnosed with DR-TB between 2011 and 2015 who were resident in the Hlabisa health subdistrict. Since 2011, all patients with DR-TB aged ⩾12 years have been admitted to the TB ward at Hlabisa Hospital for at least the first month of treatment. Since July 2013, individuals with DR-TB in the subdistrict have been identified using Xpert^®^ MTB/RIF testing (Cepheid, Sunnyvale, CA, USA) at one of the 17 primary health care clinics. Before that, most patients were identified using culture-based methods, apart from a small number of patients diagnosed using Xpert at a clinical trial site.^18^

We identified patients with DR-TB using the International Classification of Diseases, Tenth Revision (ICD-10) discharge codes in the Hlabisa Hospital information system. We defined DR-TB based on South African coding standards, incorporating codes for rifampicin-monoresistant, multidrug-resistant (MDR) and extensively drug-resistant (XDR) TB.^19^ We calculated the proportion of patients who had DR-TB in the hospital admissions data, and described the characteristics of the patients. Data from the hospital information system are routinely linked to AHRI demographic surveillance data using the South African identification number or through a standard probabilistic matching algorithm.^15^ We used linked data to identify individual homestead locations.

The study protocol was approved by the Biomedical Research Ethics Committee of the University of KwaZulu-Natal, Durban, South Africa (ref BE290/16), the Ethics Committee of the London School of Hygiene & Tropical Medicine, London, UK (ref 11814), and the Health Research Committee of the KwaZulu-Natal Department of Health, Pietermaritzburg, South Africa (ref 378/16). All committees waived the requirement for individual informed consent to use hospital admissions data, as the data were routinely collected from hospital records and there was no direct interaction with individual patients.

#### Spatial analyses

We conducted two spatial analyses, which derived the geographic locations of patients using different methods: a local area analysis covering the entire Hlabisa subdistrict, and a micro-geographic analysis using the precise locations of patient homesteads within the smaller AHRI demographic surveillance area.

In the local area analysis, we compared the spatial distribution of DR-TB patients with all other hospital admissions. Local areas are informal regions used by local populations to describe the subdistrict, and have been mapped by AHRI (315 local areas in the Hlabisa subdistrict in total, Figure 1B). We extracted patient-reported local areas of residence from a free-text field in the hospital data, and matched them to mapped names of local areas.

In the micro-geographic analysis, we compared point residential locations for patients with DR-TB with the spatial distribution of the general population. We used residential locations from the AHRI demographic surveillance data. For DR-TB patients, we identified the exact homestead of residence recorded in the surveillance system closest in time to the patient’s date of admission to Hlabisa Hospital. The distribution of the general population was derived by calculating the total person-years of residence in each homestead over the study period.

We tested for spatial clustering of DR-TB in both the local area and micro-geographic analyses. We used spatial scan statistics, implemented in SaTScan software (National Cancer Institute, Bethesda, MD, USA),^20^ to test the hypothesis that DR-TB patients were closer together in space than the underlying population distribution. Scan statistics are used to compare the observed number of cases within spatial windows of various sizes with those that would be expected, in this case under a random Poisson distribution. A likelihood ratio was calculated for each window to compare the observed and expected numbers of cases inside and outside the window. Monte Carlo simulations were then used to generate random distributions of cases under the Poisson distribution, which were compared with observed data to calculate a *P* value. We set the maximum cluster size to 3 km because spatial dependencies have previously been reported for HIV within this distance in this study area.^21^

We also plotted the locations of clusters on a smoothed map of the relative proportion of DR-TB patients compared with the underlying distribution in continuous geographic space. These maps were produced using kernel density estimation, with a standard Gaussian kernel of 3 km radius. Analyses were performed using R v3.2.3 (R Computing, Vienna, Austria) using spatstat and rsatscan packages.^22^,^23^

## RESULTS

Between 2011 and 2015, 19 408 individuals admitted to Hlabisa Hospital could be allocated to a local area in the Hlabisa subdistrict. Of these, 489 (2.5%) had an ICD-10 hospital discharge code indicating DR-TB, the majority (*n* = 478, 98%) of whom had MDR-TB disease.

Characteristics of patients with DR-TB are shown in the Table. Approximately half (*n* = 250, 51%) were female, and the modal age group was 25–34 years. There were 340 (70%) HIV-positive DR-TB patients, 202 (60%) of whom were on antiretroviral therapy (ART) at the time of admission. One in six (*n* = 78, 16%) DR-TB patients died before discharge, and five were lost to follow-up.

**Table:** Characteristics of DR-TB patients, Hlabisa Hospital, 2011–2015

Characteristic	*n* (%)
Sex	
Male	239 (48.9)
Female	250 (51.1)
Age group, years	
5–14	10 (2.0)
15–24	59 (12.1)
25–34	166 (33.9)
35–44	143 (29.2)
45–54	66 (13.5)
55–64	27 (5.5)
65–74	10 (2.0)
⩾75	8 (1.6)
Year of admission	
2011	77 (15.7)
2012	98 (20.0)
2013	103 (21.1)
2014	115 (23.5)
2015	96 (19.6)
Type of DR-TB	
MDR-TB	478 (97.8)
Rifampicin-monoresistant	5 (1.0)
XDR-TB	6 (1.2)
Site of disease	
Pulmonary	421 (86.1)
Extra-pulmonary	5 (1.0)
Missing	63 (12.9)
HIV/ART status*	
HIV-positive, on ART	202 (41.3)
HIV-positive, not on ART	133 (27.2)
HIV-positive, ART missing	5 (1.0)
HIV-negative	67 (13.7)
Missing	82 (16.8)
Discharge status	
Discharged	394 (80.6)
Transferred	12 (2.5)
Died	78 (16.0)
Lost to follow-up	5 (1.0)

*At the time of hospital admission.

DR-TB = drug-resistant tuberculosis; MDR-TB = multidrug-resistant TB; XDR-TB = extensively drug-resistant TB; HIV = human immunodeficiency virus; ART = antiretroviral therapy.

### Local area analysis of drug-resistant tuberculosis in the Hlabisa subdistrict

We used the distribution of all 19 408 patients admitted to Hlabisa Hospital across the local areas in the subdistrict as a denominator for analyses of spatial clustering among the 489 DR-TB patients.

**Figure 2.**
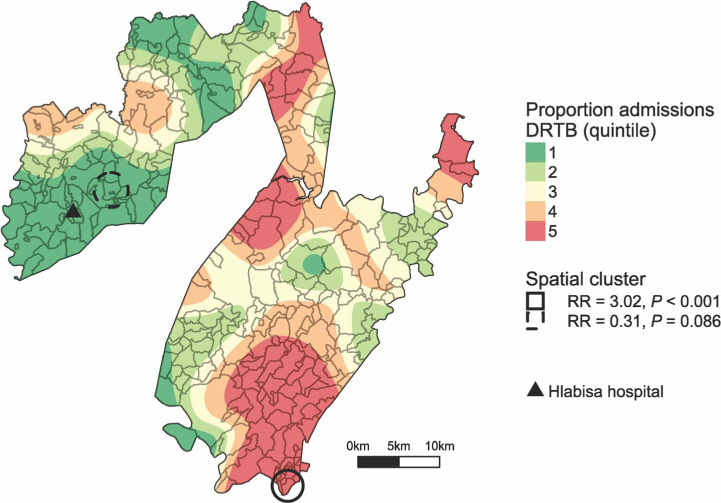
Spatial clustering of drug-resistant tuberculosis in Hlabisa subdistrict, South Africa, 2011–2015. Locations determined using patient-reported local areas in hospital information system. DR-TB = drug-resistant tuberculosis; RR = relative risk. This image can be viewed online in colour at http://www.ingentaconnect.com/content/iuatld/ijtld/2018/00000022/00000003/art00010

There was one high relative risk (RR) cluster, located in the south-east of the subdistrict (*P* <0.001). This cluster had a radius of 1.9 km, comprised four local areas, with 79 DR-TB patients compared with the 29 that would be expected by chance, and had an RR of 3.0. There was some evidence of a low RR cluster in the west of the subdistrict, close to Hlabisa Hospital (*P* = 0.08). This cluster had a radius of 2.1 km, comprised four local areas, with six patients compared with 19 expected, and an RR of 0.3. The locations of these two clusters, overlaid on a smoothed map of the relative proportion of DR-TB patients compared with all hospital admissions, are displayed in Figure 2.

### Micro-geographic analysis of drug-resistant tuberculosis in the Africa Health Research Institute demographic surveillance area

There were 144 DR-TB patients whose hospital data could be linked to the AHRI population surveillance data. Of these, 111 had a recorded homestead location during the study period and were included in the analysis: 94 patients had a residential location recorded in the same year as their admission date, 16 of the remaining patients had a residence recorded before their admission date, and one had a residence location recorded the year after the admission date. The remaining 33 patients were excluded from this analysis because they did not have a homestead of residence recorded during the period of this study (2011–2015).

**Figure 3.**
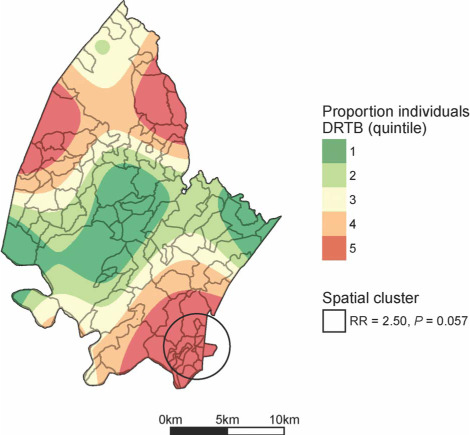
Spatial clustering of drug-resistant tuberculosis in Africa Health Research Institute demographic surveillance area, 2011–2015. Locations determined by linking hospital data to individual homesteads in demographic surveillance system. DR-TB = drug-resistant tuberculosis; RR = relative risk. This image can be viewed online in colour at http://www.ingentaconnect.com/content/iuatld/ijtld/2018/00000022/00000003/art00010

The 111 patients with DR-TB resided at 106 unique homestead locations; 10 patients shared homesteads with another patient. The most likely high RR cluster (*P* = 0.057) had a radius of 2.8 km (Figure 3). The cluster comprised 55 patients compared with 31 expected, had an RR of 2.5, and all the homesteads with more than one patient were in this area. It was in a similar region to the high-risk cluster resulting from the local area-level analysis of the entire Hlabisa subdistrict, in the south-east of the demographic surveillance area. This is the area around a township, and is characterised by high population density and HIV prevalence compared with the rest of the demographic surveillance area.^21^ No low RR clusters were identified in this analysis.

## DISCUSSION

In the present study, we have described, for the first time, the spatial distribution of DR-TB in the Hlabisa subdistrict of KwaZulu-Natal. DR-TB was highly prevalent in this region, with 489 (2.5%) of the in-patients at Hlabisa Hospital affected over a 5-year period. Almost all had MDR-TB disease and 16% died in hospital. There was clear evidence of a geographic high-risk cluster of DR-TB in the south-east of the region. This area is characterised by relatively high population density and high incidence and prevalence of HIV.^21^ This spatial heterogeneity of DR-TB in a high-burden, predominantly rural area was consistent with findings from lower HIV prevalence settings, although our analysis was at a more granular level than that in most previous studies.^6^,^10^,^11^

Establishing the spatial distribution of disease in rural areas such as the Hlabisa subdistrict is challenging. This is because residential addresses are not routinely recorded in hospital systems, many people live in informal settlements which are not accurately mapped, and the population is highly mobile.^24^ One strength of our study was that we used precise residential locations collected in the AHRI demographic surveillance system. We were therefore able to derive the geographic distribution of DR-TB from two different data sources: the self-reported local area of residence from hospital data and, for residents of the AHRI demographic surveillance area, the homestead of residence. High-risk clusters of disease were indicated in the same approximate area using both methods, suggesting that the observed clustering was genuine.

The area of spatial clustering was characterised by high population density, and only 10 (18%) patients in the cluster shared residences with other patients. This finding implies that transmission of DR-TB in this community may have occurred in public places as well as within households. Other studies have also indicated the importance of community-based transmission of TB in similar settings.^12^,^25^–^30^ Indoor venues with poor ventilation in which people come into close contact, including health care facilities, public transport, churches and bars, have been implicated as possible areas of transmission. An important component of DR-TB prevention is therefore to identify such venues in the community and implement interventions, including active case finding by regular screening, contact tracing, improving access to treatment, and undertaking measures to control airborne infection in health facilities.

The district-based spatial clustering of the disease suggests that targeting these interventions at suspected high transmission areas could be effective. However, our findings only reveal where people with DR-TB reside; uncovering precisely where transmission is occurring will require more detailed clinical and molecular epidemiology. A prospective cohort of people with DR-TB is now operational in the study area, where information is collected about social contact patterns and use of shared public spaces. This information will be integrated with whole-genome sequence data to provide a better understanding of transmission.

The results of our study also highlight the importance of the interaction between HIV and TB in this population. Almost three quarters of the patients with DR-TB were HIV-positive, compared with a population prevalence of approximately one quarter. The area of spatial clustering of DR-TB was characterised by high HIV prevalence, and is in a similar region to a geographic cluster of HIV-positive individuals identified previously by Tanser et al.^21^ In this study population, approximately 60% of HIV-positive patients were on ART. Previous studies have suggested that improved coverage of ART at both individual and community levels can contribute to a reduction in TB incidence.^31^–^33^

Our study had five main limitations. First, the analysis was restricted to DR-TB patients because we were only able to ascertain cases through hospital admissions data. Drug-susceptible TB patients are only admitted when clinically essential, whereas policy at the time of the study was for all drug-resistant patients to be admitted for at least 1 month. We were therefore unable to determine whether the distribution of drug-resistant disease was similar to that of drug-susceptible disease. Future studies in this area will integrate additional data from the electronic TB register (ETR.net) to characterise the distribution of drug-susceptible TB.

Second, the hospital information system used in our study did not contain clinical information about the history of TB treatment. With the data available, we therefore could not make any inference about the balance of primary and secondary drug resistance in the population. However, most recent data from high HIV prevalence settings suggest that, regardless of treatment history, the majority of DR-TB cases arise from transmission.^34^

The third limitation was the use of hospital discharge codes to define DR-TB patients, which is an underestimate of the true number. We will have missed patients who were not coded in the hospital data as DR-TB, children aged <12 years who may have been managed elsewhere, individuals with detected DR-TB who did not go to hospital, and those with undetected disease. However, we have no reason to suspect that these factors would operate in a geographically heterogeneous way that would lead to spurious spatial clusters.

Fourth, the spatial analysis of DR-TB in the wider Hlabisa subdistrict was limited by the use of local areas as opposed to individual addresses. However, the results were similar to the analysis of precise point locations in the demographic surveillance area, which suggests that this method may be useful for similar analyses in the future. We also used a hospital-based denominator as a proxy for the underlying population in this analysis. This will therefore be influenced by the spatial factors that govern the distribution of conditions relating to other admissions.

Finally, we described the characteristics of individuals using hospital data, but further information on patients would allow a more detailed analysis of risk factors in this population.

## CONCLUSIONS

Our study provides worrying evidence of possible ongoing transmission of DR-TB in this area of high prevalence. This observation suggests that targeting interventions to spatial areas of highest risk could be effective in supporting progress towards the WHO’s End TB strategy for a 90% reduction in new cases by 2035.^35^
